# Bracteanolide A abrogates oxidative stress‐induced cellular damage and protects against hepatic ischemia and reperfusion injury in rats

**DOI:** 10.1002/fsn3.2374

**Published:** 2021-07-22

**Authors:** Ting‐Yu Chao, Cheng‐Chu Hsieh, Yueh‐Hsiung Kuo, Ya‐Ju Yu, Cho‐Hua Wan, Shu‐Chen Hsieh

**Affiliations:** ^1^ Institute of Food Science and Technology National Taiwan University Taipei Taiwan; ^2^ Biologics Division Animal Health Research Institute Council of Agriculture Executive Yuan, New Taipei City Taiwan; ^3^ Department of Chemistry National Taiwan University Taipei Taiwan; ^4^ Department of Chinese Pharmaceutical Sciences and Chinese Medicine Resources China Medical University Taichung Taiwan; ^5^ Department of Biotechnology Asia University Taichung Taiwan; ^6^ Chinese Medicine Research Center China Medical University Taichung Taiwan; ^7^ Graduate Institute of Molecular and Comparative Pathobiology School of Veterinary Medicine National Taiwan University Taipei Taiwan

**Keywords:** antioxidant, bracteanolide A, hepatic ischemia and reperfusion injury, hepatoprotection

## Abstract

Liver diseases, including viral hepatitis, liver cirrhosis, and liver cancer, mostly remain silent until the late stages and pose a continuing threat to millions of people worldwide. Liver transplantation is the most appropriate solution in the case of liver failure, but it is associated with hepatic ischemia and reperfusion (I/R) injury which severely reduces the prognosis of the patients. In order to ameliorate I/R injury, we investigated the potential of bracteanolide A, from the herb *Tradescantia albiflora* Kunth in protecting the liver from I/R injury. We first determined the protective effect of bracteanolide A against oxidative stress and DNA damage using HepG2 hepatocyte cell line and then assessed the levels of inflammatory cytokines and antioxidant proteins in response to hepatic insult using an animal model of hepatic I/R injury. The results showed bracteanolide A greatly enhanced cell survival and decreased reactive oxygen species (ROS) production under H_2_O_2_ induction. It also upregulated the expression of nuclear factor (erythroid‐derived 2)‐like2 (Nrf2) and its downstream cytoprotective proteins NAD(P)H quinone oxidoreductase 1 (NQO1) and heme oxygenase‐1 (HO‐1). Bracteanolide A effectively reduced the severity of liver lesions in I/R‐injured rats revealed by histological analysis and significantly decreased the levels of alanine transaminase (ALT), aspartate transaminase (AST), cyclooxygenase‐2, and inflammatory cytokines interleukin (IL)‐1β and tumor necrosis factor (TNF)‐α. Bracteanolide A preconditioning effectively protected the liver from I/R damage in the animal model, and this easily applied procedure may provide a new means to ameliorate hepatic I/R injury during liver surgeries.

## Graphical abstract

Bracteanolide A, the active compound from *Tradescantia albiflora* Kunth, exhibits antioxidative and cytoprotective potentials. Moreover, bracteanolide A given prior to inducing hepatic ischemia and reperfusion in rats effectively lessens damage to the liver through lowering levels of ALT, AST, Suzuki score for histopathological lesions, and inflammatory cytokines, as well as restoring liver morphology. Bracteanolide A may be a feasible hepatoprotective agent against liver ischemia and reperfusion injury.

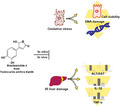

## 1 INTRODUCTION

Chronic liver disease affects millions of people and its complications, including cirrhosis and hepatocellular carcinoma, account for 3.5% of all deaths worldwide (Asrani et al., 2019; Moon et al., 2019). Apart from significant advances in vaccination and treatment of viral hepatitis, incidence of metabolic liver diseases, on the other hand, is on the rise, and this is associated with increasing alcohol and/or drug abuse and compromised health conditions such as diabetes and obesity (Xiao et al., 2019; Younossi et al., 2020). The end stage of chronic liver diseases calls for liver transplantation as a way to extend the patient's life. Liver ischemia and reperfusion injury (I/R injury) is a pathophysiological condition that occurs during and after liver resection, liver transplantation, and other surgical procedures involving the liver (Cannistrà et al., 2016). I/R injury is responsible for significant morbidity and mortality resulting from liver graft failure and multiple organ dysfunctions (Cannistrà et al., 2016). The two stages of an I/R injury, ischemia and reperfusion, affect different cells and tissues. The main cells being affected during ischemia are sinusoidal endothelial cells, which result in altered microcirculation, causing hypoxia and subsequent impaired ATP synthesis. When blood and oxygen supply is re‐established during reperfusion, the sequential activation of CD4^+^ T lymphocytes, Kupffer cells, and neutrophils releases reactive oxygen species (ROS) and inflammatory cytokines such as tumor necrosis factor‐alpha (TNF‐α), interleukin‐1 (IL‐1), and platelet‐activating factor (Konishi & Lentsch, 2017).

Inducible nitric oxide synthase (iNOS) is induced by inflammatory mediators and leads to the production of nitric oxide (NO), an initial form of reactive nitrogen species (RNS) which signals to nuclear factor (erythroid‐derived 2)‐like2 (Nrf2)/antioxidant response element (ARE) signaling, a major cellular antioxidant–detoxification mechanism in response to pathological stress (Ma, 2013; Molaei et al., 2021). NO exerts dual roles depending on dose and duration of exposure. In physiological levels, it attenuates liver I/R damage through regulating levels of ATP, cytokines, and antioxidants, but in large amounts it causes tissue damage (Guan et al., 2014). Following reperfusion, hydrogen peroxide and superoxide radicals generated through nicotinamide adenine dinucleotide phosphate (NADPH) oxidase and the xanthine‐oxidase system give rise to oxidative stress, leading to lipid peroxidation, DNA damage, and cell death (Ildefonso & Arias‐Díaz, 2010). In response to oxidative stress, Nrf2 is released from ubiquitin–proteasomal degradation and translocates to the nucleus where it binds to antioxidant response element, inducing the expression of cytoprotective genes, such as heme oxygenase‐1 (HO‐1), superoxide dismutase 1 (SOD1), NAD(P)H quinone oxidoreductase 1 (NQO1), and catalase (Wasik et al., 2017), which counterbalance oxidative stress through glutathione (GSH) regulation (Liu et al., 2017). Overexpression of HO‐1 in macrophages suppressed the production of inflammatory cytokines and preserved mouse and rat liver histology from I/R insult (Ke et al., 2010; Shen et al., 2011). Upregulation of NQO1 is found in various liver pathologies, and this may represent an adaptive stress response to halt further disease progression by detoxifying reactive species (Aleksunes et al., 2006; Cheng et al., 2015). Both Nrf2 and HO‐1 have been identified as promising therapeutic targets for the management of hepatic I/R injury (Li et al., 2019; Ma, 2013; Richards et al., 2010).

**FIGURE 1:**
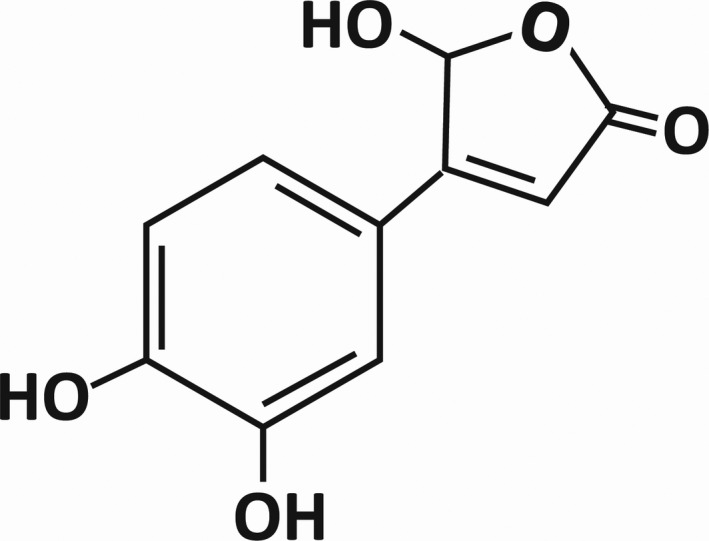
Chemical structure of bracteanolide A

Intermittent inflow occlusion and ischemic preconditioning, as well as the administration of pharmaceutical agents such as antioxidants, are current strategies used to overcome hepatic ischemia and reperfusion injury and irreversible damage (Saidi & Kenari, 2014). Given the increasing prevalence of liver illness on a global scale that ultimately requires liver transplantation or other surgical operations, which is largely susceptible to hepatic ischemia and reperfusion injury, we choose to investigate new compounds with the potential to reduce I/R damage to the liver. Bracteanolide A, whose structure is shown in Figure 1, is the major active compound of *Tradescantia albiflora* Kunth, a herb used to treat gout, edema, nephritis, enteritis, and diarrhea (Wang et al., 2016). However, evidence‐based medical efficacy of bracteanolide A is limited. Bracteanolide A is reported to be one of the main compounds in the methanol extract of *Murdannia bracteata* (MB‐m), which belongs to the same family as *T. albiflora*, and exhibits potent inhibitory effect on iNOS (Wang et al., 2007), which suggests its potential in the prevention and treatment of diseases involving an increased expression of iNOS. In addition, MB‐m possesses antioxidant and free radical scavenging activities, contributing to its protective effect against CCl_4_‐induced hepatic damage (Yam et al., 2010).

According to the abovementioned, bracteanolide A seems to be a suitable candidate as a hepatoprotective agent. Thus, we aim to investigate the potential of bracteanolide A to protect against liver damage using HepG2 cells and a rat model of hepatic ischemia and reperfusion injury.

## 2 MATERIALS AND METHODS

### 2.1 Cell culture

All materials and reagents for cell culture were obtained from Gibco, Thermo Fisher Scientific (Walham, MA, USA) unless otherwise stated. HepG2 cells were maintained in Dulbecco's Modified Eagle Medium (DMEM) containing 10% fetal bovine serum and 1% penicillin/streptomycin. DMEM supplemented with 10% fetal bovine serum, 25 mM sodium bicarbonate, 12.5 mM HEPES (Bioshop Canada Inc., Burlington, ON, Canada), 0.03% L‐glutamine, 100 mg/L sodium pyruvate, and 25 mM glucose. The cells were cultured at 37℃ in a humidified incubator containing 5% CO_2_.

### 2.2 Bracteanolide A toxicity and cell viability

The cytoxicity of bracteanolide A alone on HepG2 cells was examined using the WST‐1 assay according to manufacturer's protocol. HepG2 cells were seeded at 2 × 10^5^ per well in a 96‐well culture plate. Following incubation of bracteanolide A at 0, 5, 10, 20, 50, 100, or 200 μM for 24, 48, and 72 hr, spent medium was aspirated and WST‐1 reagent diluted 50x in cell culture medium was added to cell and incubated for 2.5 hr. Then, the culture medium was collected and absorbance OD_450–620_ was measured. Modified methylene blue assay was used to evaluate the viability of cells exposed to oxidative stress with or without bracteanolide A treatment. The method was described in Felice et al. (2009). Briefly, HepG2 cells were seeded at 8 × 10^4^ cells per well in a 24‐well plate and allowed to attach for 24 hr. For coincubation, cells were treated with vehicle or 10, 20, or 50 μM bracteanolide A together with 750 μM H_2_O_2_ for 24 hr. For preincubation, cells were first incubated with bracteanolide A at the above same concentrations for 24 hr prior to the cotreatment with H_2_O_2_. Then, media were removed and each well was rinsed with PBS. Two hundred and fifty microliters (250 μl) of methylene blue solution (1X PBS containing 1.25% glutaraldehyde and 0.6% methylene blue) was added to each well of the culture plate. After incubation at 37℃ for 60 min, the methylene blue solution was removed from the wells, and then the plates were rinsed by gentle submersion in distilled water for six times. The excess water was allowed to drain and the plate was briefly air‐dried. Four hundred microliters (400 μl) of elution solution (consisted of 1:1 ratio of ethanol:PBS containing 1% acetic acid) was added to each well. After 15 min on a plate rotator at room temperature, the elution solutions were centrifuged at 12,000 × *g* for 3 min. The solutions were transferred to a 96‐well plate in duplicates, 175 μL into each well. Plates were read at 620 nm using a microplate reader (Thermo Fisher Scientific Instruments Co. Ltd, USA).

### 2.3 Intracellular ROS assay

The generation of intracellular ROS was determined as described in Wang and Joseph (1999). Briefly, HepG2 cells were seeded at a density of 8 × 10^5^ per well in a 6‐well plate and incubated for 24 hr at 37℃. After different treatments for the indicated durations, the cells were added with 100 μM dichlorodihydrofluorescein diacetate (DCFH‐DA) (from Sigma‐Aldrich, St. Louis, MO, USA) dissolved in medium and incubated at 37℃ for 30 min. After the excessive DCFH‐DA was removed by several rinses with 1X PBS, the cells were exposed to 1 mM H_2_O_2_ for 1 hr to trigger ROS production. After lysing the cells, aliquots of the cell lysates (150 μl) were transferred to a black 96‐well fluorometric plate in duplicates. The fluorescence was measured using Thermo Scientific Fluoroskan Ascent fluorescence spectrometer (Thermo, USA). The excitation filter was set at 480 nm and the emission filter was set at 538 nm.

### 2.4 Animals and I/R model

Eighteen healthy male Sprague Dawley rats (250–300 g) were obtained from the National Laboratory Animal Center, Taiwan, Republic of China. Animals were housed two in a cage of polycarbonate material and provided with corn cobs bedding, given a standard diet and clean water ad libitum, and maintained at 23 ± 2℃, 60 ± 5% RH with adequate ventilation on a 12‐hr light/dark cycle. The experimental procedure was approved by the Institutional Animal Care and Use Committee of National Taiwan University (approval ID: NTU‐103‐EL‐91). After one week of acclimatization, rats were randomly divided into three groups: Sham, I/R, and I/R+Bracteanolide A groups. The I/R injury rat model was generated as described in Yamauchi et al. (Yamauchi et al., 1982). Briefly, each rat was anesthetized with 1%–2% isoflurane in pure oxygen. The liver was exposed through an upper midline incision, and two pieces of fine silk thread were looped along the right and left branches of the portal vein, hepatic artery, and bile duct to block blood supply to the median and left lobes. To elicit I/R injury, ischemia of the median/left lobes was maintained for 60 min after which threads were released and blood was resupplied. In the sham‐operated group (Sham), rats were given anesthesia and subjected to laparotomy as well as exposure of the portal triad without hepatic ischemia (*n* = 6). In the I/R group, rats were subjected to ischemia and reperfusion as described above (*n* = 6). In the bracteanolide A‐pretreated group (I/R+Bracteanolide A), the rats received intraperitoneal injection of bracteanolide A (2.5 mg/kg.bw), dose determined from preliminary experiment, at 30 min prior to ischemia (*n* = 6). Bracteanolide A was diluted to 0.625 mg/ml in 1% carboxymethylcellulose (Sigma‐Aldrich). The rats in Sham and I/R groups received an equivalent volume of vehicle. After ischemia, animals were sutured, wounds swabbed with iodine solution, and given baytril (10 mg/kg BW, s.c. injection), and then returned to cages provided with lamps to avoid hypothermia. Inhalation anesthesia was chosen for ease of application and monitoring of animal physiology, as well as uninterrupted duration of sufficient anesthesia. Blood pressure was monitored by tail vein sphygmomanometer throughout the operation. Signs of distress such as lethargy or reduced food intake and grooming were not observed during recovery. The rats were sacrificed 24 hr after reperfusion by anesthesia followed by cardiac puncture; blood as well as liver tissue samples were harvested for analysis.

### 2.5 Histological analysis

The left lobes of livers were harvested and trimmed to suitable sizes before being fixed in a 10% (v/v) formalin solution and then processed by standard histological routines through dehydration, clearing, and wax embedding to paraffin sections. Tissue sections (4–5 μm) were stained with hematoxylin and eosin and examined under a light microscope. Sections were scored by a pathologist from 0 to 4 for degrees of sinusoidal congestion, vacuolization of hepatocyte cytoplasm, and necrosis as described in Suzuki et al. (1991). Criteria are shown in Table 1.

**TABLE 1:** Suzuki scores for the assessment of liver damage following I/R injury

Score	Congestion	Vacuolization	Necrosis
0	None	None	None
1	Minimal	Minimal	Single cell necrosis
2	Mild	Mild	<30%
3	Moderate	Moderate	<60%
4	Severe	Severe	>60%

### 2.6 Biochemical analyses

#### 2.6.1 Serum ALT and AST levels

Blood samples were allowed to clot at room temperature for 30 min, followed by centrifugation at 3,000 × *g* for 5 min. The supernatant was collected and designated as serum. Serum alanine aminotransferase (ALT) and aspartate aminotransferase (AST) levels were measured by SPOTCHEM SP‐4410 clinical chemistry analyzer (Arkray Co, Kyoto, Japan).

#### 2.6.2 Serum cytokines

Serum levels of IL‐1β, IL‐6, IL‐10 and TNF‐α were determined using commercial assay kits (Sigma‐Aldrich, Cat. No. RAB0277, RAB0311, RAB0246, and RAB0479, respectively) according to the manufacturers’ instructions.

### 2.7 Gene expression analysis

#### 2.7.1 RNA extraction and reverse transcription polymerase chain reaction (RT‐PCR)

Total RNA in liver tissue/cell pellet was isolated using TriPure Isolation Reagent (Roche Applied Science, Basel, Switzerland). For each sample, 2 μg RNA was reverse‐transcribed using SMART MMLV reverse transcriptase (Promega, Wisconsin, USA), 10 μM triphosphate deoxyribonucleotides (dNTP), 5× first‐strand buffer, and 20 μM random hexamer primers. Reaction was performed at 70℃ for 3 min, followed by incubation at 42℃ for 60 min, and finally at 75℃ for 15 min to terminate the reaction.

#### 2.7.2 Quantitative PCR (q‐PCR)

Quantitative PCR analyses were performed on aliquots of cDNA to detect *iNOS*, *COX‐2*, *HO‐1*, *NQO1*, *Nrf2*, and *18S* (as an internal standard) gene expression using a thermal cycler (Applied Biosystems, Foster City, CA, USA). Reactions were carried out in a volume of 10 μL containing: 5 μL 2× KAPA SYBR®, 0.2 μL each of 10 μM forward and reverse primers, 2.6 μL PCR water, and 2 μL cDNA. After an initial enzyme activation for 3 min at 95℃, 40 amplification cycles were performed (3 s for 95℃ denaturation, 20 s for 60℃ annealing and extension). The primers used in this study were purchased from Sigma‐Aldrich (USA) and are listed in Table 2.

**TABLE 2:** Sequences of primers used for q‐PCR

Genes	Forward (5'−3')	Reverse (3'−5')
18S	TATTCCCATGACCCGCC	GTGAGGTTTCCCGTGTT
rat iNOS	CCAGGAGATGTTGAACTACG	CGCATTAGCACAGAAGCAAA
rat COX−2	GTCTTTGGTCTGGTGCC	TCACTATCTTGATCGTCTCTCCTA
rat HO−1	GCTCTATCGTGCTCGCATGA	AATTCCCACTGCCACGGTC
rat NQO1	ACTCGGAGAACTTTCAGTACC	TTGGAGCAAAGTAGACTGGT
human Nrf2	AGTAGGTAACTGTAGTCCACAT	GTTGCTGATACTGGGCTC
human NQO1	GCTAGGTATCATTCAACTCTCCA	AGCAAGAAATATCATAAACAAGCAT
human HO−1	AACTTTCAGAAGGGCCAGGT	CTGGGCTCTCCTTGTTGC

Abbreviations: COX‐2, cyclooxygenase‐2; HO‐1, heme oxygenase‐1; iNOS, inducible nitric oxide synthase; NQO1, NAD(P)H quinone oxidoreductase 1; Nrf2, nuclear factor (erythroid‐derived 2)‐like2.

### 2.8 Western blot analysis

After treatment with 0, 10, 20, or 50 μM bracteanolide A for the indicated times, adherent cells were collected for protein extract preparation. Briefly, HepG2 cells were lysed with RIPA buffer containing 50 mM Tris (pH 7.5), 150 mM NaCl, 1% Triton X‐100, 0.1% SDS, 1% sodium deoxycholate, protease inhibitors (2 μg/ml aprotinin, 5 μg/ml leupeptin, 1 μg/ml pepstatin A, 1 mM phenylmethylsulfonyl fluoride), 0.5% NP‐40, and 5 mM EDTA for whole‐cell lysate preparation. Nuclear extracts were isolated from cell pellet using hypertonic buffer containing 10 mM HEPES pH 7.0, 0.5 M KCl, 1 mM Mg(CH_3_COO)_2_, 1 mM DTT, 10% glycerol, 1 mM PMSF, 0.5% NP‐40, and 5 mM EDTA. Cytoplasmic extracts were separated using hypotonic buffer containing 10 mM HEPES pH 7.0, 10 mM KCl, 1 mM Mg(CH_3_COO)_2_, 1 mM DTT, 10% glycerol, 1 mM PMSF, 0.5% NP‐40, and 5 mM EDTA. Equal amounts of lysate were then electrophoresed using SDS‐PAGE, blotted onto polyvinylidene difluoride membranes, conjugated with various specific primary antibodies (NQO1, GTX100235; HO‐1, GTX101147; Nrf2, GTX103322; p‐H2AX, 2577S; beta‐actin, GTX110564; lamin B1, ab133741) at dilutions suggested on the datasheet, and then probed with appropriate HRP‐conjugated secondary antibodies. The immunoreactive bands were detected using the enhanced chemiluminescent (ECL) method and visualized on UVP BioSpectrum Imaging System (UVP CPQ 8478, Cambridge, UK).

### 2.9 Statistical analysis

Results were expressed as mean ±standard error of the mean (*SEM*). Comparisons in different groups were made using one‐way or two‐way analysis of variance (ANOVA) where appropriate followed by post hoc tests. Statistical significance was defined as *p* < .05.

## 3 RESULTS

### 3.1 Bracteanolide A protects HepG2 cells against H_2_O_2_‐induced oxidative stress

**FIGURE 2:**
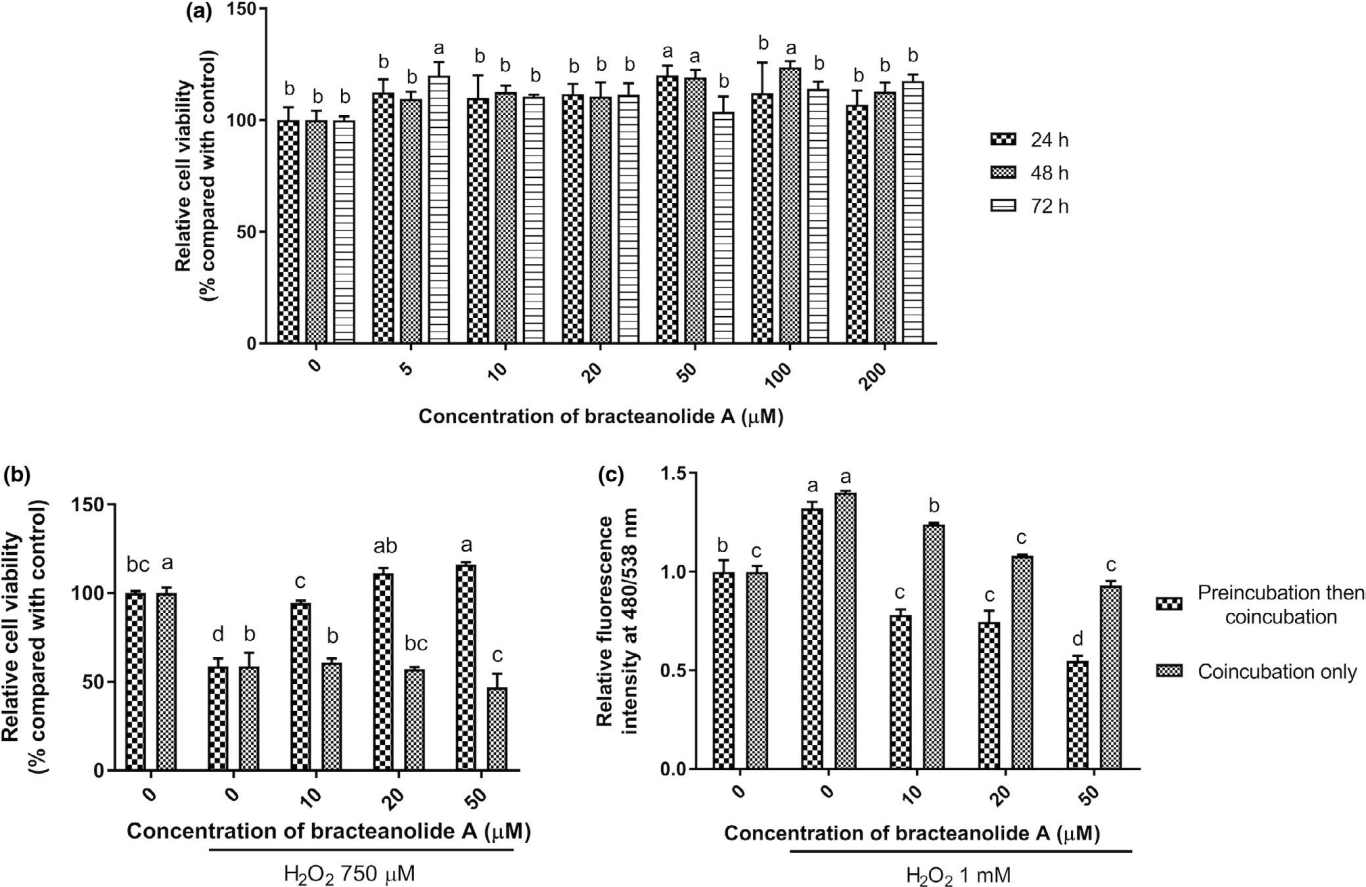
Effects of bracteanolide A on cell viability and reactive oxygen species production under H2O2 induction. (a) Cells were treated with bracteanolide A at 5, 10, 20, 50, 100, and 200 μM for 24, 48, or 72 hr and then harvested to test for cytotoxicity of bracteanolide A on the cells using WST‐1 assay. (b) Cells were treated with 750 μM H2O2 and bracteanolide A at 0, 10, 20, and 50 μΜ for 24 hr, with or without pretreatment of bracteanolide A at the same concentrations for 24 hr. Modified methylene assay was then performed to estimate cell viability. The relative percentage of viability to control cells (set as 100%) is shown as mean±*SEM* from at least two independent experiments. (c) Cells were treated with 1 mM H2O2 and bracteanolide A at the concentrations indicated for 1 hr, without or without pretreatment of bracteanolide A at the same concentrations for 24 hr. The ROS concentration was measured by fluorescence at ex 480/em 538 nm. Differences among various concentrations within each treatment duration (a) or the two incubation groups (b and c) were analyzed by two‐way ANOVA followed by Tukey's multiple comparisons test. Different letters represent statistical significance at *p* <.05

We first examined the cytoxicity of bracteanolide A on HepG2 cells (from the hepatocyte lineage) using WST‐1 assay. The result shows that bracteanolide A does not induce cytotoxicity at all the concentrations treated from low to high doses (Figure 2a) and for all three treatment durations (24, 48, and 72 hr). Then we determined whether bracteanolide A can protect HepG2 cells against oxidative stress to assess its potential reduce ischemia/reperfusion injury to the liver. Figure 2b shows that H_2_O_2_ significantly decreased the cell viability of HepG2, while pretreatment, but not cotreatment, of bracteanolide A for 24 hr significantly increased the survival of HepG2 cells exposed to oxidative stress in a dose‐dependent manner. In addition, bracteanolide A significantly sequestrated the intracellular reactive oxygen species as detected by the amount of fluorescence from 2’,7’‐dichlorofluorescein (DCF), which was upregulated upon H_2_O_2_ induction. Similar to the trend observed in cell survival rate, preincubation can accentuate bracteanolide A’s ROS scavenging effect compared to coincubation (Figure 2c).

### 3.2 Bracteanolide A induces nuclear translocation and the protein expression but not mRNA levels of Nrf2

**FIGURE 3:**
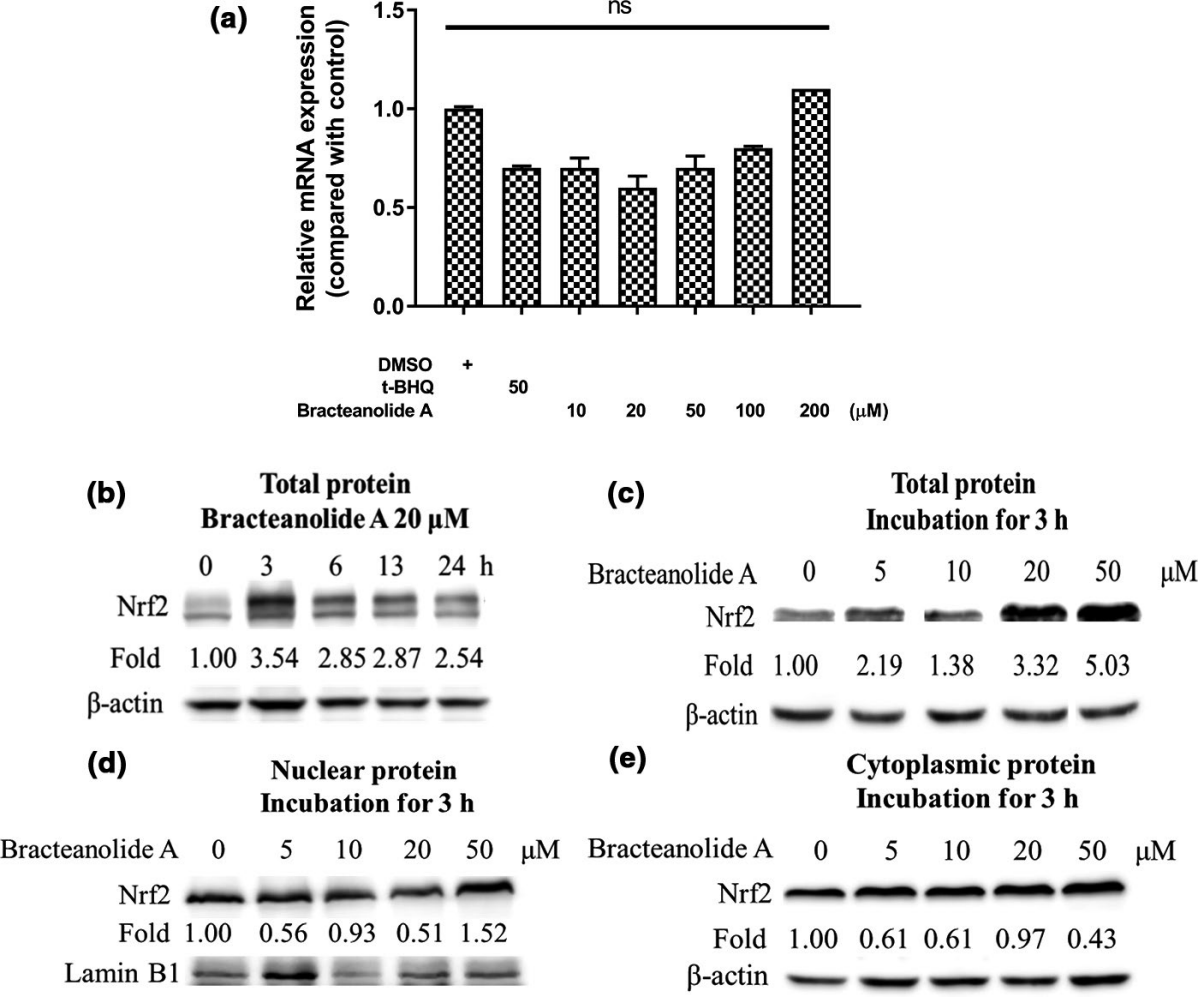
Effects of bracteanolide A on cellular Nrf2 expression (a) mRNA expression of *Nrf2* in HepG2 cells treated with bracteanolide A at 0, 10, 20, 50, 100, and 200 μM for 17 hr. Equal amount of total RNA extracts from different treatments was reverse‐transcribed and cDNA was quantified by qPCR with Nrf2 specific primers. 18S was used as an internal control. Representative result is expressed as mean±*SEM* from at least two independent experiments. ns =not significant. (b and c) Nrf2 protein levels were assessed from cells treated with 20 μM bracteanolide A for 0, 3, 6, 13, and 24 hr (b) and from cells treated with bracteanolide A at 0, 5, 10, 20, 50 μM for 3 hr (c). (d and e) Nrf2 translocation under 0, 5, 10, 20, and 50 μM bracteanolide A incubation for 3 hr was assessed by nuclear protein fraction (d) and cytoplasmic protein fraction (e). Equal amount of protein extracts was separated on SDS‐PAGE and analyzed by Western blot with the Nrf2 antibody. β‐actin was used as a loading control. Representative results are shown from at least two independent experiments

To investigate the underlying reason for bracteanolide A’s protection against H_2_O_2_‐induced oxidative stress and ROS production, we looked into changes of Nrf2, a key regulator in the cellular antioxidant response. First, we measured time‐course Nrf2 mRNA expression using tert‐butylhydroquinone (t‐BHQ), a common antioxidant food additive in fats and oils, as a positive control for inducing Nrf2. We found 17 hr treatment can induce a significant increase compared to shorter or longer durations; thus, 17 hr was used as the treatment duration for mRNA expression in subsequent experiments involving NQO1 and HO‐1. Then, we treated cells with increasing concentrations of bracteanolide A from 0, 10, 20, 50, 100, and 200 μM and found out there was no significant difference among the various doses (Figure 3a), suggesting bracteanolide A did not regulate Nrf2 at the gene level, which is not surprising for a transcription factor. Later, we went on to investigate whether bracteanolide A regulates Nrf2 at the protein level. We incubated cells with 20 μM bracteanolide A for different durations to find out the most suitable time for Nrf2 induction, which was 3 hr as shown in Figure 3b. Thus, cells were then incubated for 3 hr in different concentrations of bracteanolide A. The results (Figure 3b–d) showed Nrf2 protein induction was most evident at 50 μM bracteanolide A (Figure 3c), which induced a 5.03‐fold increase in Nrf2 total protein and nuclear protein accumulation, indicating Nrf2 activation. Subsequently, 50 μM bracteanolide A was the dose used to investigate protein expression of Nrf2 downstream targets NQO1 and HO‐1.

### 3.3 Bracteanolide A stimulates the expression of Nrf2‐mediated cytoprotective enzymes, NQO1 and HO‐1 in HepG2 cells

**FIGURE 4:**
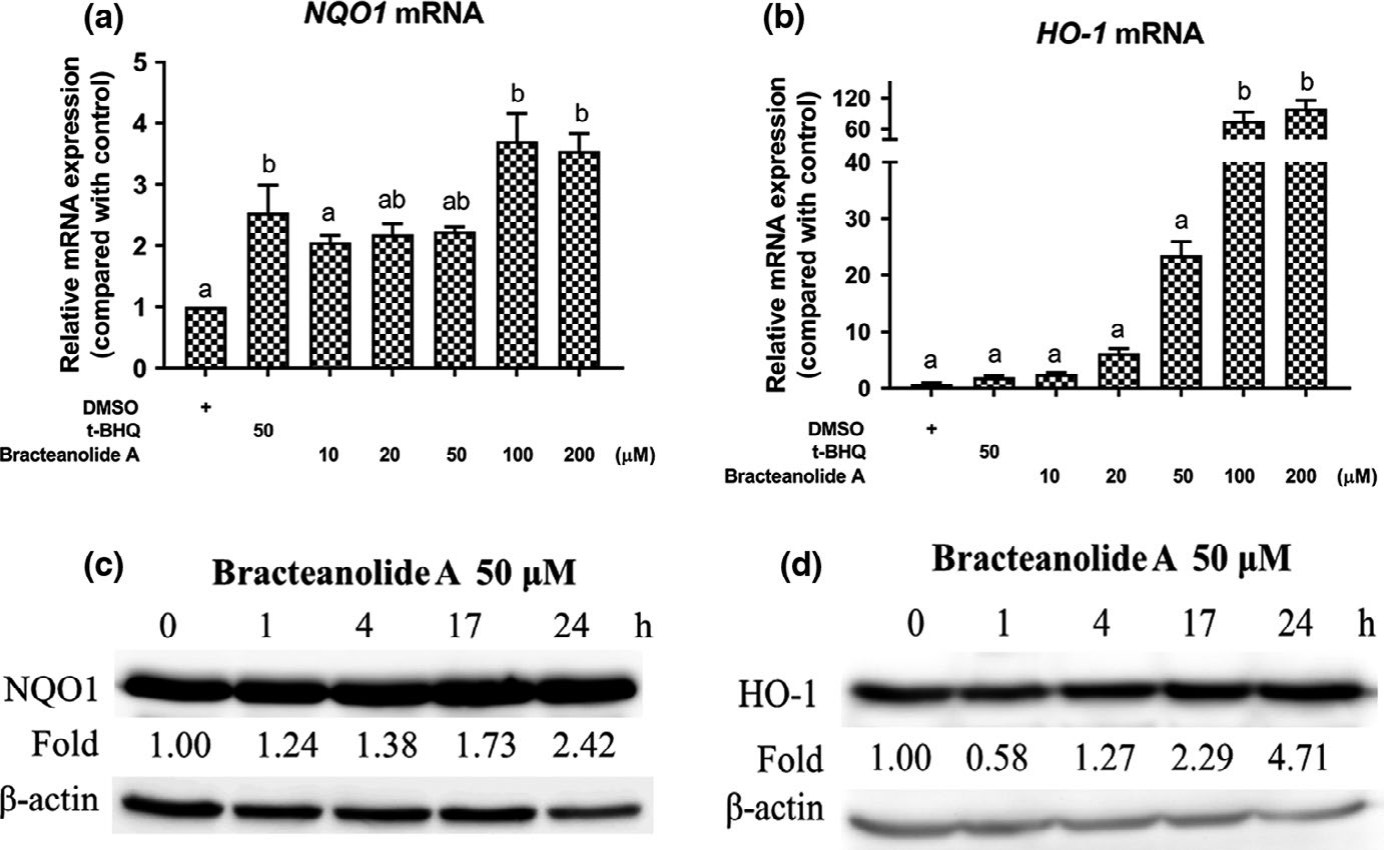
Effects of bracteanolide A on cellular NQO1 and HO‐1 expression (a) The effect of bracteanolide A on the mRNA expression of *NQO1* or (b) *HO‐1* at the indicated concentrations in HepG2. Cells were treated with bracteanolide A at the concentrations 0, 10, 20, 50, 100, and 200 μM for 17 hr. Equal amount of total RNA extracts was reverse‐transcribed and then cDNA was quantified by qPCR with primers for NQO1 or HO‐1. 18S was used as an internal control. Different letters indicate statistical significance at *p* <.05 compared to the control (DMSO at 0.1% v/v) group. Representative result is expressed as mean±*SEM* from at least two independent experiments. (c) Protein expression of NQO1 and (d) HO‐1 in HepG2 cells treated with 50 μM bracteanolide A for the indicated durations. Equal amounts of protein extracts were separated by SDS‐PAGE and analyzed by Western blot with the NQO1 or HO‐1 antibody. β‐actin was used as a loading control. Representative result is shown from at least two independent experiments.

NQO1 and HO‐1 are transcriptional targets of Nrf2; therefore, we examined the changes in NQO1 and HO‐1 expression under bracteanolide A treatment. Figure 4a,b shows the mRNA expression of NQO1 and HO‐1 increased significantly at 100 and 200 μM. In addition, 24‐hr incubation with 50 μM bracteanolide A, which was sufficient to induce Nrf2 nuclear translocation, increased NQO1 protein by 2.42‐fold and HO‐1 protein by 4.71‐fold (Figure 4c,d).

### 3.4 Bracteanolide A protects HepG2 cells from oxidative stress‐induced DNA damage

**FIGURE 5:**
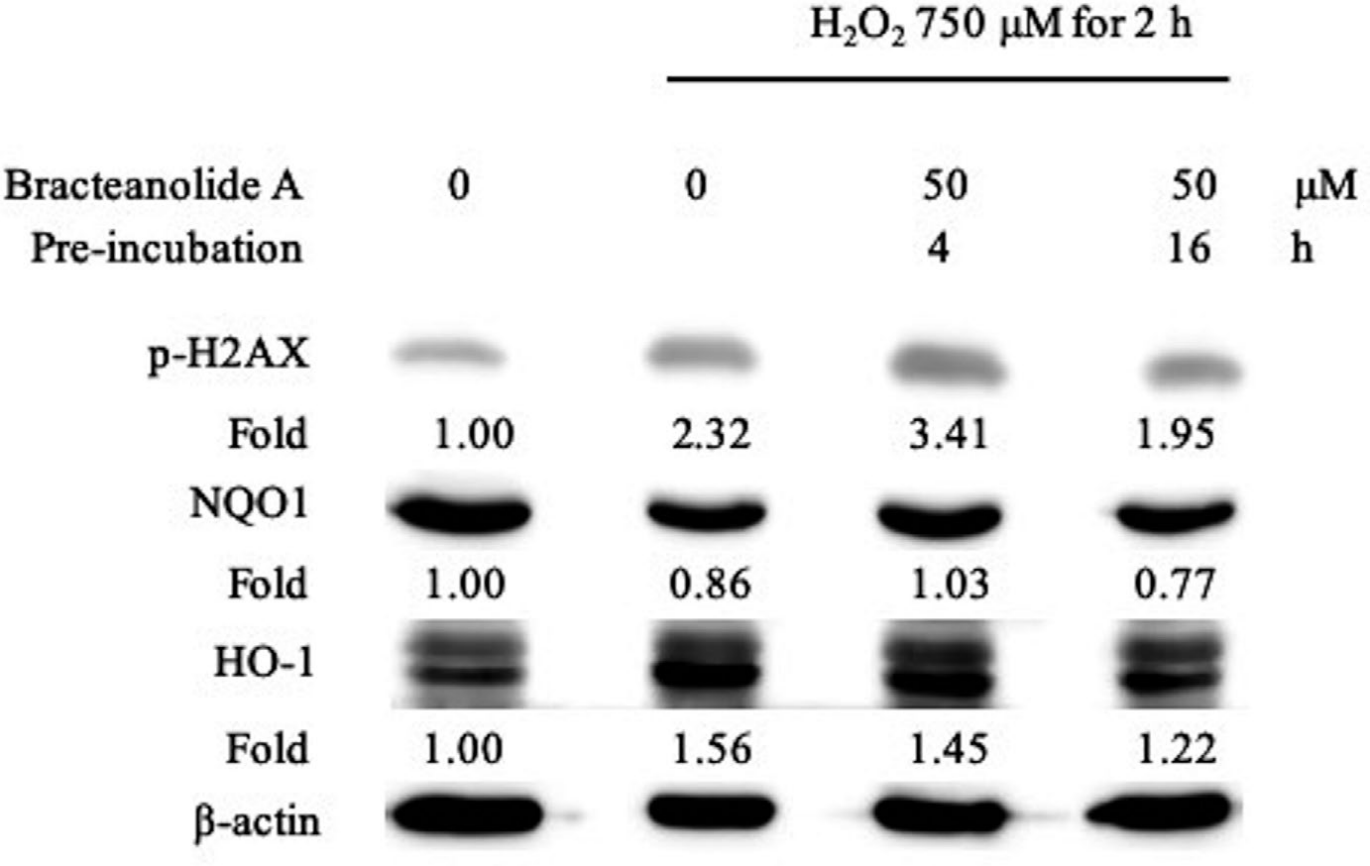
Effect of bracteanolide A on p‐H2AX protein expression (DNA damage). Cells were pretreated with 50 μM bracteanolide A for 4 or 16 hr, followed by 750 μM H2O2 for 2 hr. The expression pattern of phosphorylated H2AX (p‐H2AX) along with NQO1 and HO‐1 was analyzed by Western blot with p‐H2AX, NQO1, and HO‐1 antibodies, respectively. β‐actin was used as a loading control. Representative data are shown from at least two independent experiments

Phosphorylation of H2AX is an early biomarker for DNA double‐strand breaks (Kuo & Yang, 2008). Apart from protecting cells from oxidative stress‐induced cell death by abrogating ROS production and stimulating the levels of cytoprotective proteins, bracteanolide A also reduced DNA damage caused by H_2_O_2_. As shown in Figure 5, preincubation of bracteanolide A for 4 hr could not rescue H_2_O_2_‐induced increase in p‐H2AX expression. Bracteanolide A preincubation for 16 hr, however, reduced the expression of p‐H2AX, with a concomitant decrease in NQO1 and HO‐1 levels.

### 3.5 Bracteanolide A ameliorates I/R‐induced hepatic damage

**FIGURE 6:**
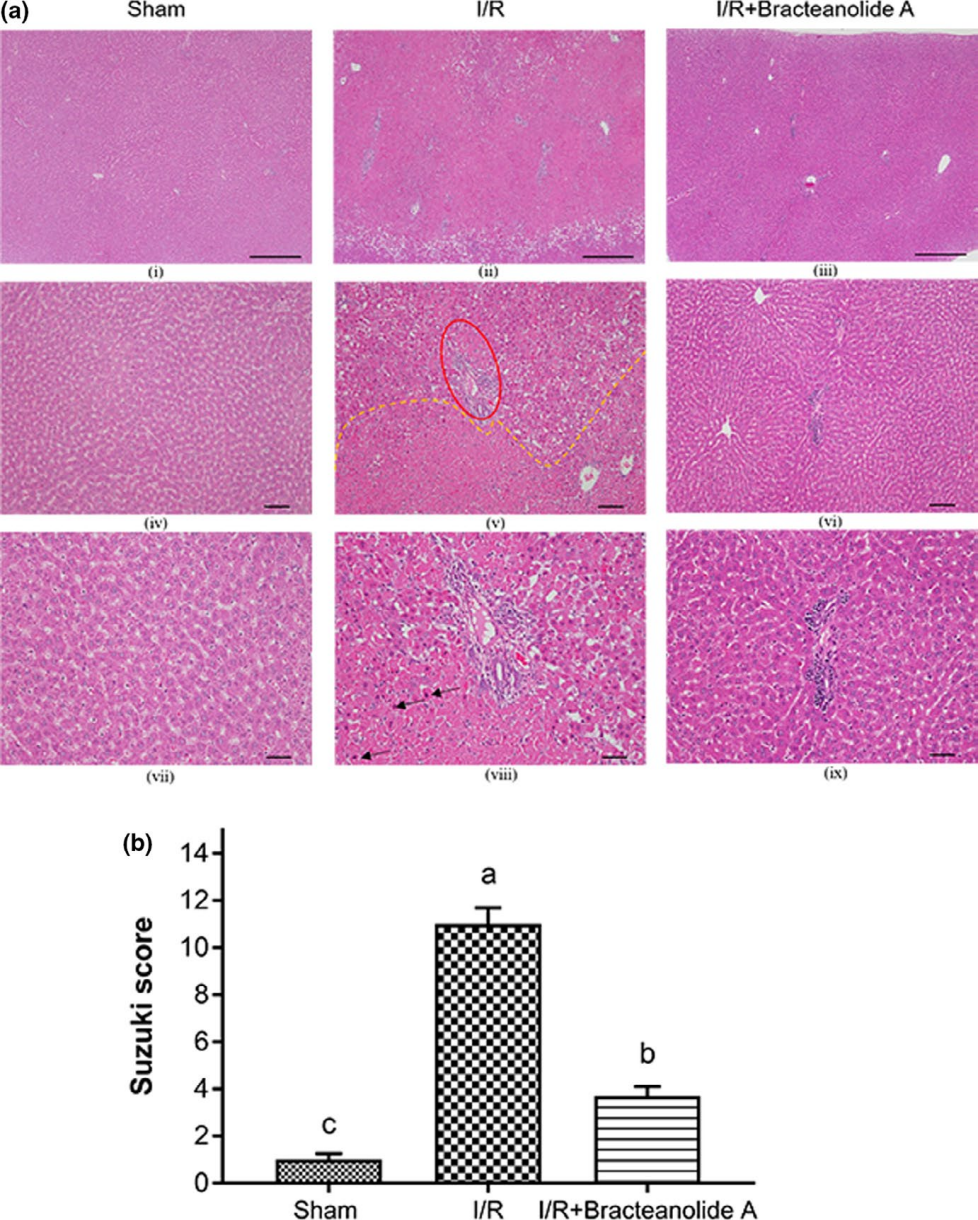
Effects of bracteanolide A on rat liver pathological lesions and Suzuki score. (a) Histological analysis of livers in sham rats (i, iv, vii), I/R rats (ii, v, viii), and bracteanolide A‐treated I/R rats (iii, vi, ix). Compared to Sham, I/R group exhibits large areas of necrotic cells (ii and area below yellow dashes in v) and periportal neutrophilic inflammation with mild lymphocyte infiltration (red oval in v), as well as pycnosis (black arrows in viii), while I/R+Bracteanolide A shows in general, healthy cells, albeit slight peribiliary lymphocytic infiltration. Microphotographs taken at 40X (i, ii, iii, scale =500 μm), 100X (iv, v, vi, scale =100 μm) and 200X (vii, viii, ix, scale =50 μm) magnification. (b) Suzuki scores on the liver sections graded histologists based on the severity of lesions according to the scoring scheme published by Suzuki and colleagues shown in Table 1. Data are shown as mean±*SEM*. *N* = 6. Different letters indicate statistical significance at *p* <.05

**FIGURE 7:**
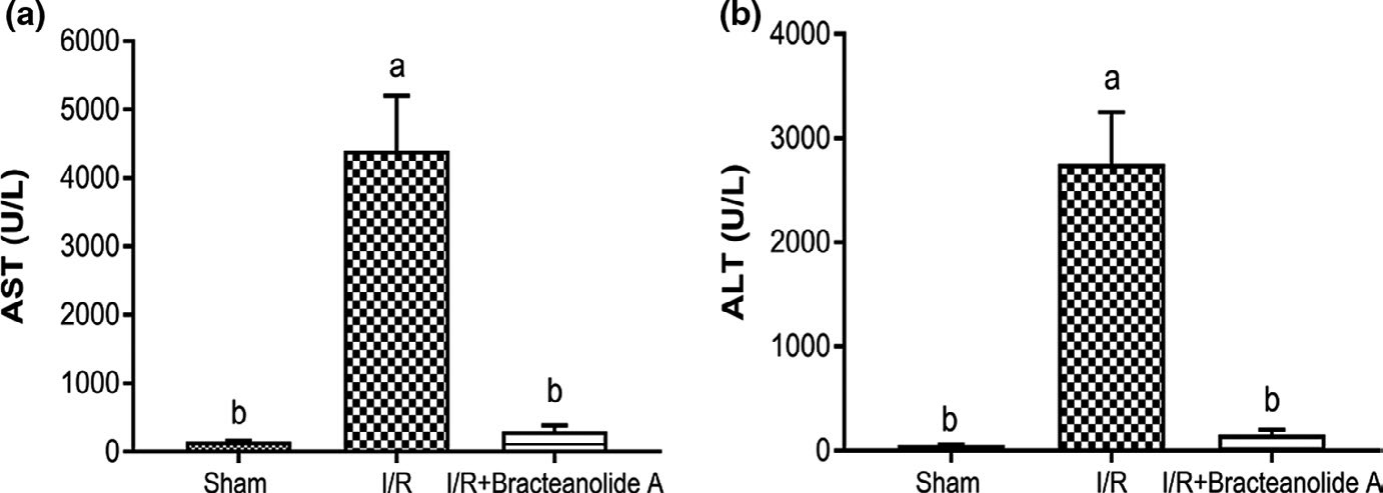
Effects of bracteanolide A on serum AST and ALT. (a) Aspartate aminotransferase (AST) and (b) alanine aminotransferase (ALT) activities in serum were measured. Rats were given bracteanolide A at 2.5 mg/kg body weight by intraperitoneal injection, at 30 min prior to hepatic ischemia. Twenty‐four hour after reperfusion, rats were sacrificed and serum samples were isolated and tested for levels of AST and ALT. Data are shown as mean±*SEM*. *N* = 6. Different letters indicate statistical significance at *p* <.05

In order to further investigate the potential of bracteanolide A, we tested its effects on a rat model of hepatic ischemia and reperfusion injury, which is associated with oxidative and inflammatory insult, causing damage to the liver. We successfully reproduced distinct morphological and structural alterations to the liver by occlusion of blood supply to the median/left lobes for 1 hr followed by 24 hr of reperfusion as a model of I/R injury. Liver histological sections show normal liver parenchyma (Figure 6a i,iv,vii) and a large area of coagulative necrosis and periportal neutrophil infiltration in I/R‐injured liver (Figure 6a ii,v,vii). Bracteanolide A pretreatment at 30 min before I/R greatly reduced cellular necrosis, inflammatory infiltration as well as significantly decreased the Suzuki score (grading scheme shown in Table 1) compared to I/R rats (Figure 6a iii, vi, ix and 6b). Moreover, bracteanolide A also significantly reduced the plasma levels of ALT and AST, markers of liver damage, which soared in I/R‐injured rats (Figure 7).

### 3.6 Bracteanolide A restores I/R‐induced alterations in the mRNA levels of inflammatory and cytoprotective enzymes

**FIGURE 8:**
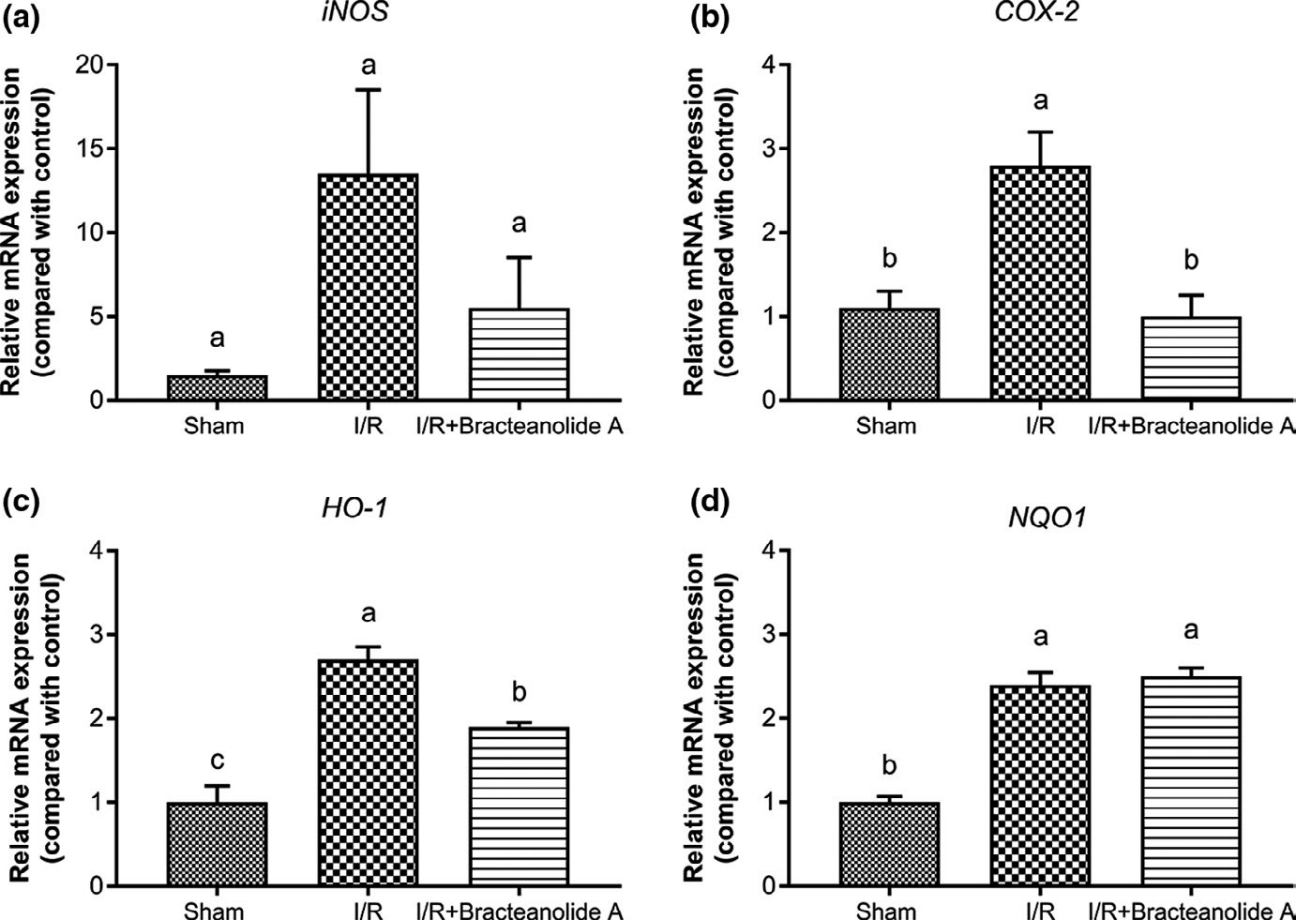
Effects of bracteanolide A on hepatic mRNA levels of inflammatory markers and cytoprotective. Enzymes. Effects of ischemia/reperfusion and treatment with bracteanolide A on mRNA expression of (a) inducible nitric oxide synthase (iNOS), (b) cyclooxygenase‐2 (COX‐2), (c) heme oxygenase‐1 (HO‐1), and (d) NAD(P)H quinone oxidoreductase 1 [NQO1] in liver tissue. Procedures are described in Materials and Methods. Data are shown as mean±*SEM*. *N* = 6. Different letters indicate statistical significance at *p* <.05

It was clear that bracteanolide A pretreatment significantly ameliorated I/R injury as shown by improvement in morphology and structure of the liver and significant reduction in ALT and AST levels. We went on to look at the levels of inflammatory indices iNOS and COX‐2 as well as Nrf2‐mediated cytoprotective enzymes NQO1 and HO‐1. Hepatic I/R significantly increased the mRNA levels of iNOS and COX‐2, as well as those of HO‐1 and NQO1. Administration of bracteanolide A significantly suppressed COX‐2 and HO‐1 levels, but not iNOS and NQO1 (Figure 8b,c).

### 3.7 Bracteanolide A decreases serum levels of inflammatory cytokines

**FIGURE 9:**
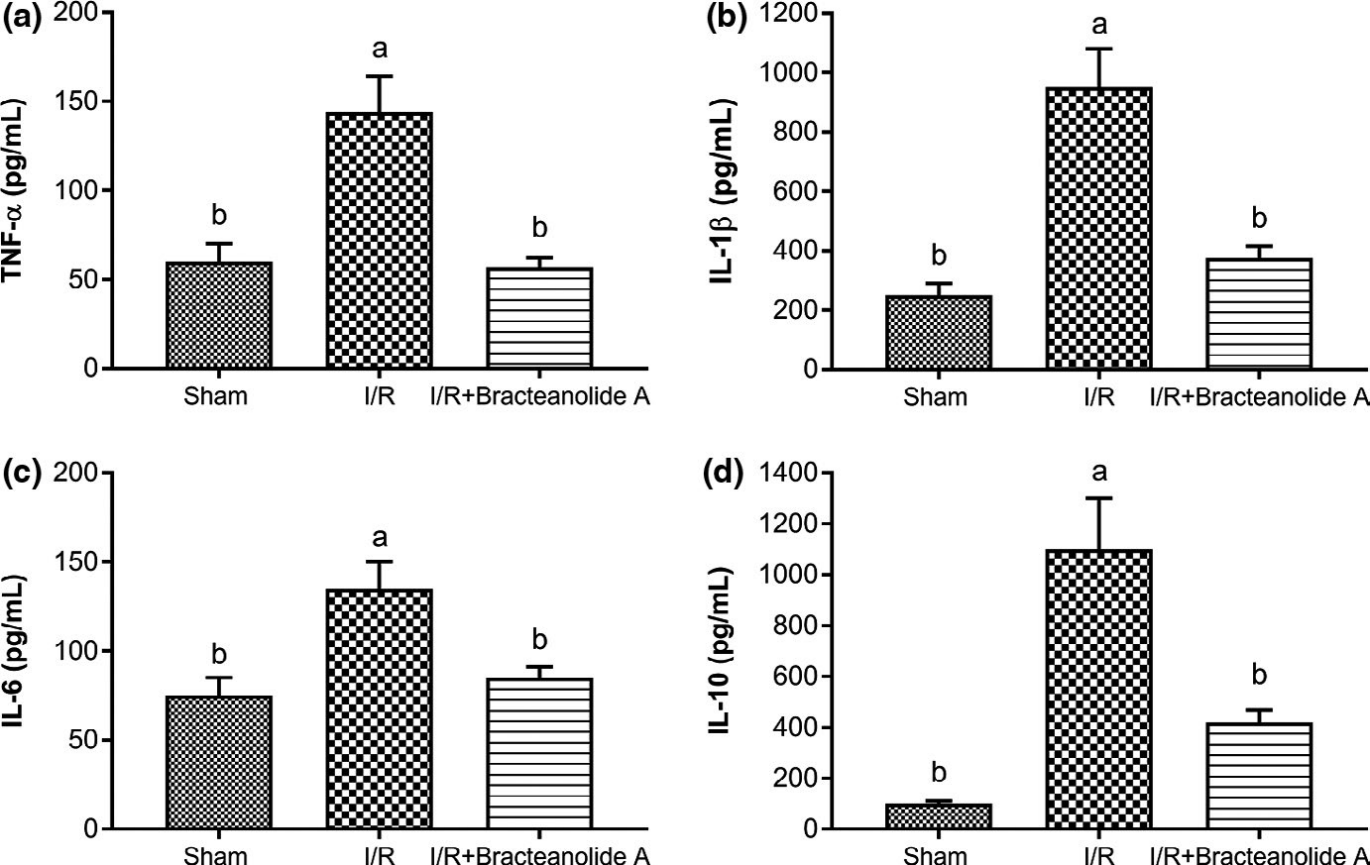
Effects of bracteanolide A on cytokine levels in the serum of I/R‐injured rats. Effects of ischemia/reperfusion and treatment with bracteanolide A on (a) interleukin 1β (IL‐1β), (b) interleukin 6 (IL‐6), (c) interleukin 10 (IL‐10), (d) tumor necrosis factor‐α (TNF‐α) levels in rat serum. Data are shown as mean±*SEM*. *N* = 6. Procedures are described in Materials and Methods. Different letters indicate statistical significance at *p* < .05

Serum inflammatory cytokines were significantly increased upon I/R insult, as shown in Figure 9. However, consistent with the reduction in COX‐2 mRNA levels, bracteanolide A administration effectively reduced the levels of the inflammatory cytokines, even to levels comparable to Sham rats.

## 4 DISCUSSION

Plant extracts contain phytochemicals which are active in preventing various diseases and may be beneficial to health with moderate consumption. Many studies have explored the potential of plant extracts against liver injuries (Park et al., 2020; Wang et al., 2019; Zhu et al., 2019). The pathology of I/R‐induced hepatic injury reveals that oxidative stress plays a crucial role in the scenario. The Keap1/Nrf2 pathway is an essential cellular defense mechanism against reactive oxygen species (Baird & Dinkova‐Kostova, 2011; Kobayashi & Yamamoto, 2006), making it rational to assess Nrf2 expression levels in our study. To find a preconditioning candidate that can activate Nrf2, we first screened compounds with antioxidant potential through a luciferase‐based platform (data not shown). Bracteanolide A came out to be the most promising agent among the candidates investigated; this result was confirmed by bracteanolide A’s potent protection against H_2_O_2_‐induced cell death in this study (Figure 2b). Furthermore, bracteanolide A was revealed to be an effective ROS scavenger, significantly inhibiting the production of intracellular ROS with pretreatment (Figure 2c). Usually, transcription factors were regulated at the post‐translational level (Filtz et al., 2014), which is consistent with our finding that without influencing the mRNA level of Nrf2, bracteanolide A increased Nrf2 protein expression most at 3 hr and further intensified with increasing doses (Figure 3a–c), indicating bracteanolide A regulated Nrf2 at the protein level. Moreover, nuclear concentration of the Nrf2 protein increased at 50 μM of bracteanolide A, while the cytoplasmic concentration steadily decreased with increasing doses, confirming Nrf2 nuclear translocation where it controls the expression of downstream targets (Figure 3d.e). Previous studies have shown Nrf2/NQO/HO‐1 signaling to be upregulated as an adaptive response to oxidative stress (Liu et al., 2018; Loboda et al., 2016; Thekkeveedu et al., 2018). Indeed, both the mRNA and protein expression of NQO1 and HO‐1 were enhanced by bracteanolide A treatment, suggesting bracteanolide A induces Nrf2 activation and subsequent induction of NQO1 and HO‐1 (Figure 4), thus ameliorating cell death as a mechanism of defense upon H_2_O_2_‐induced oxidative insult (Figure 2b).

In the present animal study, we used 60 min of median/left lobar ischemia followed by 24 hr of reperfusion as a model of I/R injury. In consistence with the result observed in the cell model, bracteanolide A showed a protective effect on I/R‐induced alterations in liver histology and biochemical parameters in the blood (Figures 6 and 7). Levels of HO‐1 and NQO1 mRNA were upregulated as a normal defense in response to I/R‐induced oxidative insult; upon Bracteanolide A treatment, HO‐1 mRNA was lowered (Figure 8c). Cytokines such as IL‐1β, produced by Kupffer cells and hepatocytes, can upregulate leukocyte aggregation and adhesion, as well as NO production through protein kinase B (Akt), NF‐κB, and iNOS pathways. The upregulation of NF‐κB increases TNF‐α and IL‐6; TNF‐α in turn causes the overproduction of chemokines and ROS, leading to activation of T lymphocytes and neutrophil accumulation that aggravate liver injury (Cannistrà et al., 2016). In this study, bracteanolide A triggered a significant reduction of I/R‐induced increase in COX‐2 mRNA levels (Figure 8b) and a concurrent decline in IL‐1β and TNF‐α levels (Figure 9a.b), showing bracteanolide A effectively attenuated the propagation of I/R injury. IL‐6 and IL‐10, however, have been reported as protective factors that reduce liver damage and promote tissue regeneration (Abu‐Amara et al., 2010). The observation that they were upregulated during I/R and then returned to basal level by bracteanolide A may suggest the amelioration of the hepatic insult due to the intervention. Finally, in addition to the amelioration of inflammation, we assume that a consequence of oxidative stress, DNA damage, was also prevented by bracteanolide A. It has been well established that histones wrap around DNA strands to form nucleosomes and play a central role in transcription regulation, DNA repair, DNA replication, and chromosomal stability (Paull et al., 2000). Detection of γH2AX, formed by phosphorylation of the histone variant H2AX at Ser‐139, is considered a specific and sensitive marker of DNA damage and repair (Dickey et al., 2009; Mah et al., 2010; Podhorecka et al., 2010). Whereas accumulation of γH2AX compromises cell survival, 16‐hr preincubation of bracteanolide A effectively reduced H_2_O_2_‐induced DNA damage by decreasing the protein expression of γH2AX (Figure 5).

## 5 CONCLUSION

We reproduced pathophysiological alteration characteristic of hepatic ischemia and reperfusion injury in rats by median/left lobar ischemia for 1 hr followed by 24 hr of reperfusion as a model. Our results conclude that the pretreatment of bracteanolide A attenuates oxidative stress through activating Nrf2, the key transcription factor in the cytoprotective regulatory machinery and its downstream effectors, NQO1 and HO‐1. Following Nrf2 induction, ROS‐ and inflammatory‐mediated damage is diminished and hepatic I/R injury in rats is effectively minimized, as shown by the restoration of normal structure and function of the liver. The results of this study provided evidence to bracteanolide A’s protective effect on I/R‐induced liver damage, and preconditioning with bracteanolide A may be a novel means to reduce the severity of I/R injury to hepatic tissues and to improve the clinical outcome of patients going through surgical operations on the liver.

## CONFLICT OF INTEREST

The authors declare that they do not have any conflict of interest.

## AUTHOR CONTRIBUTIONS

**Ting‐Yu Chao:** Formal analysis (equal); Investigation (equal); Methodology (supporting); Visualization (equal); Writing‐original draft (lead); Writing‐review & editing (lead). **Cheng‐Chu Hsieh:** Conceptualization (equal); Data curation (equal); Formal analysis (lead); Investigation (lead); Methodology (equal); Writing‐original draft (supporting). **Yueh‐Hsiung Kuo:** Conceptualization (equal); Funding acquisition (supporting); Investigation (supporting); Methodology (supporting); Resources (supporting); Writing‐review & editing (supporting). **Ya‐Ju Yu:** Data curation (equal); Formal analysis (equal); Investigation (equal); Methodology (equal); Visualization (equal); Writing‐original draft (supporting). **Cho‐Hua Wan:** Formal analysis (equal); Funding acquisition (equal); Investigation (equal); Project administration (supporting); Supervision (equal); Writing‐review & editing (equal). **Shu‐Chen Hsieh:** Conceptualization (equal); Funding acquisition (equal); Investigation (equal); Project administration (equal); Supervision (equal); Writing‐review & editing (equal).

## ETHICAL STATEMENT

All procedures regarding animal study and design were approved by the Institutional Animal Care and Use Committee of National Taiwan University (IACUC No. NTU‐98‐EL‐109) and carried out according to national and university guidelines.

## CONFLICT OF INTEREST

This study does not involve any human subjects.
